# Predicting Recurrence of Non-Muscle-Invasive Bladder Cancer: Current Techniques and Future Trends

**DOI:** 10.3390/cancers14205019

**Published:** 2022-10-14

**Authors:** Aya T. Shalata, Mohamed Shehata, Eric Van Bogaert, Khadiga M. Ali, Ahmed Alksas, Ali Mahmoud, Eman M. El-Gendy, Mohamed A. Mohamed, Guruprasad A. Giridharan, Sohail Contractor, Ayman El-Baz

**Affiliations:** 1 Biomedical Engineering Department, Faculty of Engineering, Mansoura University, Mansoura 35516, Egypt; 2 Bioengineering Department, University of Louisville, Louisville, KY 40292, USA; 3 Department of Radiology, University of Louisville, Louisville, KY 40202, USA; 4 Pathology Department, Faculty of Medicine, Mansoura University, Mansoura 35516, Egypt; 5 Computers and Control Systems Engineering Department, Faculty of Engineering, Mansoura University, Mansoura 35516, Egypt; 6 Electronics and Communication Engineering Department, Faculty of Engineering, Mansoura University, Mansoura 35516, Egypt

**Keywords:** NMIBC, recurrence, AI-based prediction systems, markers

## Abstract

**Simple Summary:**

Non-muscle-invasive bladder cancer is associated with its high rates of progression and recurrence, the proper diagnosis and management can save lives. Bladder cancer is the tenth most common type of cancer in the world. This review discusses the current markers used in the recurrence prediction of non-muscle-invasive bladder cancer and future trends, published in the last decade, in addition to the limitations and future prospects in the field of AI-based prediction systems.

**Abstract:**

Bladder cancer (BC) is the 10th most common cancer globally and has a high mortality rate if not detected early and treated promptly. Non-muscle-invasive BC (NMIBC) is a subclassification of BC associated with high rates of recurrence and progression. Current tools for predicting recurrence and progression on NMIBC use scoring systems based on clinical and histopathological markers. These exclude other potentially useful biomarkers which could provide a more accurate personalized risk assessment. Future trends are likely to use artificial intelligence (AI) to enhance the prediction of recurrence in patients with NMIBC and decrease the use of standard clinical protocols such as cystoscopy and cytology. Here, we provide a comprehensive survey of the most recent studies from the last decade (N = 70 studies), focused on the prediction of patient outcomes in NMIBC, particularly recurrence, using biomarkers such as radiomics, histopathology, clinical, and genomics. The value of individual and combined biomarkers is discussed in detail with the goal of identifying future trends that will lead to the personalized management of NMIBC.

## 1. Introduction

According to GLOBOCAN 2020, bladder cancer (BC) has the 10th highest incidence among cancers globally [1]. By 2022, in the United States, two out of ten patients with BC are expected to die from the disease [2,3]. Bladder cancer is usually classified into two main categories, non-muscle-invasive bladder cancer (NMIBC) and muscle-invasive bladder cancer (MIBC). The majority of bladder tumors are non-muscle-invasive and include the following pathological stages: papillary tumors confined to bladder mucosa (Ta) which account for most NMIBC cases, invasion of the subepithelial connective tissue (stage T1), and tumor in situ (Tis), which is a high-grade, non-invasive urothelial carcinoma. Approximately 50% of patients with NMIBC will progress to MIBC if they go untreated, and the recurrence rate after treatment is approximately 70%–80% [4,5]. Bladder cancer accounts for almost USD 3.7 billion in direct costs in the US [6]. The treatment costs for NMIBC range from USD 5594 to USD 9554 per patient [7]. The cost of care varies considerably depending on the stage at first diagnosis and the success of initial treatment. The global burden of the disease is rising drastically [8,9] as the availability of some treatments, such as BCG instillation, is decreasing [10]. This reinforces the need for accurate tools which can predict treatment success at early stages.

Several diagnostic techniques can effectively detect and predict the recurrence of NMIBC. Such techniques can also suggest an optimal treatment plan for patients at first diagnosis. Current surveillance relies on the gold standard methods of cystoscopy, urine cytology, imaging, and biopsy (usually via the transurethral resection of bladder tumor or TURBT). Newer endoscopic techniques, such as white light cystoscopy, promise to improve the detection of first or recurrent NMIBC [11]. Imaging modalities as multi-slice spiral CT, MRI, transurethral ultrasound of the bladder, and positron emission tomography/computed tomography (PET/CT) can increase the diagnostic accuracy and provide useful pre-procedural information before TURBT is performed [12,13,14]. FDG PET/CT, in particular, has a high prognostic value in assessing patients with suspected recurrent BC [15,16]. MRI has recently become an important tool in the management of BC, giving rise to VI-RADS (Vesical Imaging-Reporting and Data System), a standardized reporting system aiming to improve the management of BC. [17,18]. Biopsy and histology are valuable tools for the initial diagnosis of NMIBC or the evaluation of recurrence and can improve management by determining the tumor stage and grade [3]. Recently, combinations of radiomic, clinical, pathological, and genomic markers have been used for the prediction of recurrence and to improve risk stratification [19,20].

To personalize the management of the disease, efforts have been directed toward the development of more effective tools for risk stratification, the prediction of recurrence, and the selection of optimal treatment. Previous risk assessment tools, including WHO 1973 and WHO 2004/2016 classification systems, European Organization for Research and Treatment Club (EORTC), and the Club Urologico Español de Tratamiento Oncologico (CEUTO), used clinical and histopathological markers to stratify patients into low, intermediate, and high risks of progression or recurrence [21]. The mainstay treatment for high- and intermediate-risk patients identified following TURBT is the local instillation of Bacillus Calmette–Guerin (BCG) immunotherapy [4]. For low-risk tumors (low-grade Ta), the treatment is TURBT optionally followed by intravesical chemotherapy with mitomycin C, epirubicin (Ellence), or doxorubicin (Adriamycin). For patients who do not respond to BCG immunotherapy or certain patients with high-risk disease, treatment may require radical cystectomy followed by chemotherapy and radiation [4,8,22,23]. Proper grading and staging are mandatory for the selection of initial treatment, however, current predictive tools have poor accuracy for predicting recurrence. They may underestimate the recurrence and progression in low-risk NMIBC but overestimate recurrence and progression in high-risk disease [24]. In addition, several questions remain unanswered: what is the recurrence probability cut-off that justifies a certain procedure such as cystectomy or BCG treatment? Can the type and dose of intravesical treatment be optimized based on a more precise determination of the recurrence risk? To answer such questions, new methods are imperative.

### AI in BC Management

The current generation of predictive tools for NMIBC risk assessment are based on statistical methods. In recent years, artificial intelligence (AI) has shown superior accuracy in the prediction of disease recurrence and progression compared to statistical methods. AI, a broad category of computational methods designed to mimic human intelligence, has been widely used in the medical field, usually for computer-aided diagnosis (CAD) or, in our case, computer-aided predictive (CAP) systems. A major branch of AI, machine learning (ML), was developed to solve problems in the field of medicine [25,26]. The training of an ML model or algorithm is usually divided into three steps: training, validating, and testing. During training and validation, the ML model adapts to input data and provides the outputs used for classification or regression. Both approaches fall under the umbrella of supervised learning, where the input data have been previously labeled into desired categories, representing the “ground truth”. Most medical ML applications, including those applied to NMIBC, are designed for classification using supervised learning [27].

As the management of NMIBC heavily depends on accurate diagnosis risk assessment, many CAD and CAP systems have been developed using different ML algorithms such as support vector machines (SVM), random forest (RF), artificial neural network (ANN), and deep learning (DL) [28]. The CAD systems aid in detecting BC tumors [29,30,31], bladder segmentation [32], and the identification of NMIBC [33,34]. In addition, tumor staging and grading are important factors in the personalized management of NMIBC, and have been approached using AI [35,36,37,38]. The prediction of survival rates [39], response to certain chemotherapies [40,41,42], and recurrence rates [43,44,45] are examples of CAP systems. To improve the prediction of recurrence and risk stratification, combinations of radiomic, clinical, pathological, imaging, and genomic markers have been used [19,20] to develop ML algorithms.

Here, we provide a comprehensive survey of studies from the last decade that used a wide range of markers coupled with artificial intelligence and machine learning algorithms to predict the recurrence of NMIBC at an early stage—namely clinical, radiomic, histopathological, genomic, and/or combinations of the these markers—using search engines including Google Scholar, PubMed, and ResearchGate.

We identified studies specifically related to the recurrence of NMIBC published in highly ranking journals and conferences in the last decade from 2012 to 2022. We used the following keywords individually or combined in our search: Bladder Cancer, NMIBC, Prediction, Artificial Intelligence, Machine Learning, Computer-Aided Prediction, Radiomic Markers, Clinical Markers, Histopathological Markers, Genomics, Genetic Markers, Prognostic, Recurrence, Outcomes, Surveillance, Predictor, etc., resulting in a total of 69 studies that met our inclusion criteria. Priority was given to the studies that met the aforementioned inclusion criteria and used AI or ML. To our knowledge, there is no reliable AI algorithm that can precisely predict NMIBC recurrence and the improve management of NMIBC through a combination of the aforementioned markers. We hope that, by reviewing the aforementioned studies, we open the pathway for researchers to develop highly accurate AI-based NMIBC recurrence prediction systems and provide optimal personalized management of early-stage NMIBC.

**Figure 1:**
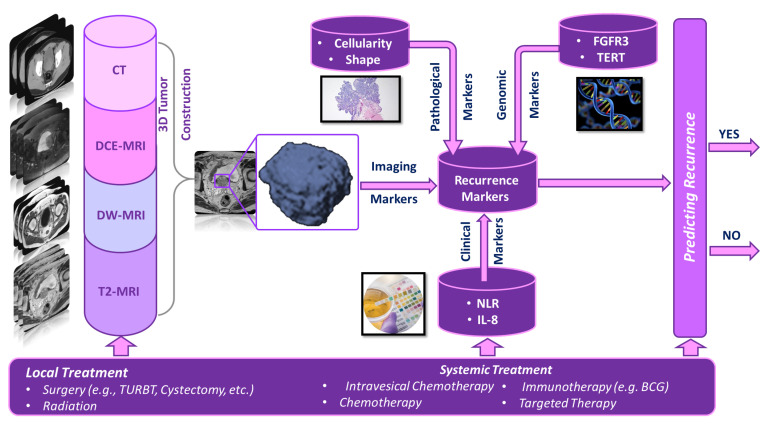
A typical pipeline for a computer-aided prediction (CAP) system that can predict the recurrence of bladder cancer at an early stage.

Below, we summarize the studies that utilized different types of predictive markers coupled with AI and ML to develop a computer-aided prediction (CAP) system (shown in Figure 1 to predict the recurrence of NMIBC. Due to the low number of studies that use AI or ML, we also considered statistical models with high accuracy. These summaries are tabulated in Tables 1–5, in which Table 1 summarizes four studies where radiomic markers were extracted from different imaging modalities; Table 2 summarizes seven studies that used histopathological slides to extract pathological markers; Table 3 summarizes 14 studies that used different clinical markers. Table 4 summarizes 25 studies where genomic markers were extracted; and Table 5 summarizes 19 studies that used combinations of the aforementioned markers.

## 2. Radiomics Markers

Several studies, as shown in Table 1, differentiate tumor from other tissues through radiologic imaging to predict recurrence. Wang et al. [46] and El-Assmy et al. [47] assessed the usefulness of different MRI sequences to predict the presence of recurrent tumors. Both studies concluded that diffusion-weighted imaging (DWI) provides the best results with an accuracy of 0.926 and 0.915, respectively. Yang et al. [48] and Alongi et al. [15] used FDG PET/CT images to predict tumor recurrence but found a lower accuracy of 0.886 for [48] and 0.90 for [15] when compared to the MRI-based studies. All four of these studies heavily relied on the experience of the physician interpreting the images and used statistical methods as opposed to AI.

The studies included in Table 1 were only concerned with differentiating recurrent NMIBC from other tissues and did not explore the power of radiomic markers as predictors of recurrence after initial treatment. To date, no AI algorithms have used only radiomic markers as the input. The four studies used the MRI signal intensity or FDG uptake on PET-CT as the most significant marker, with an accuracy ranging from 0.915 to 0.926 and from 0.886 to 0.90, respectively. Moreover, these were retrospective studies with a small number of patients. Additional markers that have the potential to improve prediction performance are illustrated below.

## 3. Histopathological Markers

Many of the studies shown in Table 2 used similar pathological parameters and immunohistochemical (IHC) markers to predict recurrence, specifically features related to tumor stage and grade. Chen et al. [49] used Ki67, a nuclear protein indicating the extent of cell proliferation, and vascular endothelial growth factor (VEGF) immunoactivity as indicators of tumor grade. Specifically, high values for both Ki67 and VEGF indicated a higher tumor grade and higher risk of recurrence. Studies by Li et al. [50], Xu et al. [51], and Zhao et al. [52] found histological variants that were predictors of poor prognosis, including squamous differentiation, glandular differentiation, and lymphovascular invasion (LVI). Chamie et al. [53] found that the tumor stage correlated with prognosis. The aforementioned studies relied on statistical regression analysis where all pathological data were pathologist-dependent and subject to interobserver variability. To address these limitations, several studies have used AI to extract features from histological slides and predict prognosis. The study by Urdal et al. [54] constructed a RUSBoost classifier with an accuracy of 0.72. Tokuyama et al. [44] compared RF and SVM-based models, finding the highest accuracy (0.90) with the SVM. The relatively higher accuracy of this algorithm may be due to a larger dataset, more textural markers, and more complete images compared to Urdal et al. [54].

Although there are few AI studies using pathological parameters and IHC markers only, they have shown that it is possible to extract textural features without the need for time-consuming human segmentation and classification [44,54] and achieve promising results with an accuracy of up to 0.90. Other studies using statistical methods [50,51,52] have identified an important variant histology which predicts poor response to intravesical therapy and suggests that earlier cystectomy could improve survival in such patients. Tumor multiplicity, tumor size, tumor grade, and tumor stage are attributed to higher morbidity and poor response to treatment [50,51,52,53]. Although Ki67 and VEFG were found to predict recurrence, the value of those predictors remains unclear since these findings have not been validated [49]. All of the studies in Table 2 included patients who underwent TURBT followed by intravesical chemotherapy, however, some of them also included other treatments, such as BCG, which could represent a confounding factor when directly comparing the results between studies. All of these studies were performed as retrospective data analyses, some with small datasets.

## 4. Clinical Markers

Clinical markers are important in current risk stratification models and in the selection of the proper treatment strategy. Pretreatment markers can help determine the need for cystectomy in high-risk patients or BCG intravesical therapy in intermediate-risk patients. Mano et al. [55] studied high-risk NMIBC patients using statistical methods to find clinical markers correlated with tumor recurrence. They found that a neutrophil-to-lymphocyte ratio (NLR) > 2.43 was associated with a high tumor grade and stage, implying a high risk of recurrence. This study was limited by an uneven distribution of stage Ta and T1 tumors in the patient groups. Rubinstein et al. [56] developed a decision tree (DT) algorithm to predict the tumor recurrence in high-grade T1 patients treated with BCG using age and NLR as independent predictors. Different accuracies were obtained from two individual cohorts and from a combination of both. The highest accuracy was found in cohort 1, with the DT model suggesting an NLR > 2.5 as the decision node, then NLR < 2.3, and lastly age > 78. Albayrak [57] suggested adjusting the age before considering NLR as a predictor. The population of this study was comprised of NMIBC patients in a very early phase, limiting the generalizability of the results. The study by Ferro et al. [58] evaluated NLR, the erythrocyte sedimentation rate (ESR), and modified Glasgow prognostic score (mGPS) as predictors of recurrence. The mGPS scoring system is classified according to C-reactive protein (CRP) levels as follows: score 0 for patients with CRP <10 mg/L without high-serum albumin levels, score 1 for patients with CRP (>10 mg/L), and score 2 for CRP (>10 mg/L) with hypoalbuminemia (<3.5 g/dL). They found that ESR, NLR, and score 1 mGPS to be predictors for the recurrence of high-grade stage T1 patients. This study was limited by its retrospective design, and all of the above studies were limited by a lack of standardized treatment.

Several urinary biomarkers have been studied as predictors of disease recurrence. Srougi et al. [59] prospectively evaluated PAI-1 and IL-8 as diagnostic markers and predictive markers of recurrence, respectively. Optimized cut-off values found using the Youden index were PAI-1 < 0.266 and IL-8 < 0.047. The analysis using logistic regression found the stability of urinary biomarker levels regardless of the use of intravesical BCG. Although the accuracy in predicting recurrence was 0.793, the specificity was poor, suggesting that the model could falsely predict lower recurrence rates than expected. Another limitation is that data were only collected early in the treatment, and so, the correlation of serial biomarkers with recurrence was not studied. Rosser et al. [60] studied 10 biomarkers including IL8, MMP9, MMP10, SERPINA1, VEGFA, ANG, CA9, APOE, SERPINE1, and SDC1. The authors developed 11 ROC models using the Wilcoxon rank-sum test to identify the association between each individual biomarker and the presence of recurrence. The best resulting model included 10 combined biomarkers with an accuracy of 0.84. The model only using the SER-PINA1 marker had the best individual marker model with an accuracy of 0.78. Limitations of this study were a small dataset and heterogeneous data with relatively few low-grade tumors.

Chevalie et al. [61] was the first prospective study to show the relationship between immunobiomarkers and recurrence in patients undergoing BCG therapy. Specifically, T-cell and monocytic myeloid-derived suppressor cells (M-MDSCs) levels were assessed. A ratio between both markers indicative of type 2 immunity was found to be a potential predictor of recurrence and predict the response to BCG therapy. The study had a small number of patients, and the results needed further validation. Alberice [62] evaluated different metabolite urinary markers: Nε, Nε-trimethyllysine, N-acetyltryptophan, dopaquinone, leucine, and hypoxanthine. Elevated levels of dopaquinone, leucine, and hypoxanthine were associated with an increased risk of recurrence for high-risk patients (TaG3 and T1G2/3). Nε, Nε-trimethyllysine and N-acetyltryptophan were associated with an increased risk of recurrence for low-risk patients Ta/G1/2. This study was limited by a small dataset and inhomogeneous patient distribution.

Several studies demonstrated the influence of cystoscopic methods on patient outcomes. For example, Naselli et al. [63] found that the use of narrow-band imaging during initial transurethral resection can decrease the risk of recurrence by at least 10% compared to white-light imaging. The primary limitation of this study was that the patients were not randomized. Work by Sfakianos et al. [64] suggested that a second restaging TURBT performed prior to BCG therapy is associated with lower rates of 5-year recurrence for high-risk patients. This study was limited by a lack of randomization. A recent study by Culpan et al. [65] evaluated the impact of delayed follow-up cystoscopy on tumor recurrence for NMIBC patients after TURBT due to the influence of the global COVID-19 pandemic. Multivariate logistic regression analysis confirmed that a 2–5 month delay is a significant risk factor in all EAU risk categories. The total number of recurrences and cystoscopy delay time were also significant risk factors for progression. Notably, no survival analysis was performed due to the limited follow-up.

A recent network meta-analysis study by Lu et al. [66] evaluated the superiority of various intravesical monotherapies in reducing recurrence in intermediate-to-high-risk NMIBC. Using a Bayesian model, they found gemcitabine to be the most effective, followed by interferon and BCG. A random-effect meta-analysis by Uhling et al. [67] found a higher recurrence rate in females compared to males, and a poor response to BCG therapy in high-risk female patients. The limitations of this study include heterogeneous data and the inclusion of only NMIBC patients receiving local treatment. A prospective study by van Osch [68] studied the effect of smoking cessation on the risk of NMIBC recurrence and found that smoking cessation was not associated with a reduced risk of recurrence. However, the treatment (smoking cessation) was not randomized and only a small proportion of patients quit during the follow-up period.

Briefly, the 22 distinct clinical markers in Table 3 were studied with the aim of predicting bladder cancer recurrence. NLR was evaluated in four studies [55,56,57,58], one of which used an AI in the form of a DT algorithm, with accuracies ranging from 0.638 to 0.923. It was also found that the age may affect the accuracy of NLR [57], and age-correction may be necessary when using NLR as a predictor of recurrence. The mGPS score and ESR [58], type of intervesical monotherapy [66], and gender [67] were found to correlate with an increased recurrence risk. Furthermore, a total of 12 urinary biomarkers were evaluated [59,60,61] with the combination of IL8, MMP9, MMP10, SERPINA1, VEGFA, ANG, CA9, APOE, SERPINE1, and SDC1 biomarkers [60] considered to be the best predictive model of the three studies. Four markers related to cystoscopy and surgical techniques were evaluated in three studies [63,64,65], including narrow-band versus white-light TURBT, restaging TURBT prior to BCG, and delays in surveillance cystoscopy. It should be noted that the included patient risk categories were highly variable between these studies, and most were retrospective, non-randomized analyses. The paucity of studies using AI suggests the potential of further personalizing patient treatment using such models. Genomic markers have shown greater success in improving NMIBC management. In the following Table 4, we illustrate a number of examples with promising results.

## 5. Genomics Markers

Currently, cystoscopy and cytology are standard tools used to monitor NMIBC, but cost-effective non-invasive tests have been developed with the aim of reducing treatment costs and improve patient follow-up. The following tests were primarily designed to detect promoter genes in urine or serum. To illustrate this, Kinde et al. [69] investigated a telomerase reverse transcriptase (TERT) promoter mutation as a significant marker for an increased risk of recurrence in NMIBC. The study showed high sensitivity compared to specificity with an accuracy of 0.933. However, the study included a very small number of patients. Likewise, Rachakonda et al. [70] found that TERT and rs2853669 polymorphisms were associated with tumor recurrence but were not statistically analyzed for accuracy, sensitivity, or specificity, despite the relatively large number of subjects. The TERT mutation, fibroblast growth factor receptor (FGFR3) gene, and OTX1 genes were evaluated as markers of increased recurrence risk by Beuker et al. [71]. However, the results were dependent on the tumor grade. Specificity was better for predicting the recurrences of high-grade than low-grade NMIBC due to a correlation between FGFR3 and tumor grade. No relation was found between the TERT mutation and tumor grade in any of the aforementioned studies [69,70,71]. Kandimalla et al. [72] studied mutations in eight genes detected in urine samples and found the highest sensitivity (74%) in a combination of OTX1, ONECUT2, and OSR1. The addition of FGFR3 increased the sensitivity to 79%. A urine-based test called UroMonitor developed by Batista [73] and colleagues detected TERT and FGFR3 mutations to detect the recurrence of NMIBC. When combined with cystoscopy, the test can detect recurrence with an accuracy of 0.90 and high specificity/sensitivity. One study that deserves mentioning is a retrospective study by Park et al. [74], which found no significant utility for FGFR3 in managing T1G3 NMIBC.

Several mRNA urine-based tests have also been developed. The CXbladder test created by Kavalieris et al. [75] analyzed the expressions of five genes: IGFBP5, HOXA13, MDK, CDK1, and CXCR2. The test result was calculated through a logistic regression model using the most recent tumor status (primary or recurrent), time of last tumor (RFS), and the five mRNA genes. The test-negative rate of this test is 0.34 with 0.93 sensitivity. The results are robust to the effects of BCG, making the test good for ruling out recurrence in intermediate-to-high-risk patients undergoing BCG therapy. An additional mRNA urine-based test, Xpert Monitor, detects ABL1, ANXA10, UPK1B, CRH, and IGF2 mRNA markers and has been validated in two studies [76,77]. Van Valenberg et al. [76] found the Xpert Monitor to have the highest sensitivity in detecting low-grade tumors and Ta recurrent tumors compared to urine cytology and UroVysion (a FISH-based urine test discussed below). Using linear discriminate analysis (LDA), an optimal accuracy of 0.79 and sensitivity of 0.74 were achieved. However, the study did not a have long-term follow-up to further validate the results. Elsawy et al. [77] verified the superiority of Xpert Monitor over urine cytology in high-grade tumors, finding a sensitivity of 100%. They also found Xpert Monitor to be an independent predictor of recurrence in patients with negative cystoscopy findings. The small number of recurrent high-grade tumors in the study (9 out of 181 patients) indicates that further testing on a larger set of patients is required to confirm the validity of the Xpert Monitor. The utility of RNA genomes was evaluated by Bi et al. [78]. They found a high correlation between low circular RNA (circRNA) expression and the risk of recurrence as well as an association with the tumor stage and grade. Lian et al. [79] found eight long non-encoding RNA (lnc-RNA) sequences (APCDD1L-AS1, FAM225B, LINC00626, LINC00958, LOC100996694, LOC441601, LOC101928111, and ZSWIM8-AS1) to be highly correlated with tumor recurrence. These studies included both MIBC and NMIBC cases, and did not report measures of test accuracy [78,79].

Additional urine-based tests include the UroVysion, a multi-target test which uses fluorescence in situ hybridization (FISH) to predict recurrence in intermediate- and high-risk patients undergoing BCG therapy. Liem et al. [45] evaluated UroVysion in three different time intervals: pre-BCG, 6 weeks post-TURBT, 3 months post-TURBT. They found a significant correlation between the recurrence and positive UroVysion test in the 3 month post-TURBT time interval with an accuracy of 0.77 and sensitivity of 0.59, noting a limited number of subjects for this interval. Kojima et al. conducted a prospective study [80], and found that two consecutive UroVysion tests predicted recurrence better than urine cytology, with an accuracy of 0.703 and 0.50 sensitivity for a single test. The UroVysion test, however, is expensive and has a high false-positive rate which could lead to increased follow-up cystoscopy and a further increased cost. Another urine-based DNA genome test, EpiCheck (BE), detected 15 DNA methylation biomarkers (Witje et al. [81]). The test had an accuracy of 0.883 and sensitivity of 0.971 when the low-grade tumors were excluded. Test accuracy was not affected by current or previous treatments. In a prospective study, Roupret et al. [82] evaluated the ADXBLADDER urine-based which uses the MCM5 DNA gene status as a single marker and found an accuracy of 0.688, a sensitivity of 0.449, and an NPV of 0.99. A urine-based NMP22 gene immunoassay (Önal et al. [83]) was found to be superior to urine cytology in the overall cohort and in low-grade tumors, but with lower sensitivity and specificity than urine cytology for high-grade tumors. The overall sensitivity and specificity of the NMP22 assay were 0.854 and 0.765, respectively. The author concludes that using NMP22 with cytology is an optimum predictive solution for recurrence.

The levels of DNA methylation in specific markers can also be used to detect the recurrence of NMIBC. Three DNA methylation markers, including SOX1, IRAK3, and L1-MET, were tested by Su et al. [84]. These markers outperformed cytology and cystoscopy in early recurrence detection with a sensitivity of 0.86 and a specificity of 0.80. Shindo et al. [85] evaluated four miRNA methylation markers (iR-9-3, miR-124-2, miR-124-3, and miR-137) collected from urine samples at the time of recurrence and during a follow-up period. Using the number of methylated genes (M-score), they found a sensitivity of 0.62 and a specificity of 0.74 for current recurrence. An M-score ≥3 was correlated with worse recurrence-free survival. A study of five DNA methylation markers by Reinert et al. [86] included HOXA9, POU4F2, TWIST1, VIM, and ZNF154. VIM had the highest sensitivity (0.89) and specificity (1.0). The authors noted that combining FGFR3 mutation analysis with DNA methylation analysis could increase the sensitivity for the detection of recurrence. Maldonado et al. [87] analyzed promoter methylation in the CCND2, CCNA1, and CALCA genes in urine samples and found statistically significant differences between patients with recurrent and non-recurrent tumors as well as significant differences between patients with BC and controls. Bellmunt et al. [88] evaluated nine markers (RHOB, ARID1A and TP53 mutations, CDKN2A deletion, and focal gain in CCNE1, PVRL4, YWHAZ, E2F3-SOX4, and PPARG genes) for association with recurrence and progression in patients with high-grade T1 NMIBC. They found significant correlations with disease progression, recurrence, as well as good outcomes among the various markers. A study by Kobayashi et al. [89] found significant correlations between human leukocyte antigen (HLA) genotypes in serum samples and intravesical recurrence after BCG therapy. Specifically, the combination of HLA-B07 and HLA-B44 homozygosity with CUETO is significantly correlated with intravesical recurrence. A meta-analysis by Galeshoot [90] searched for single nucleotide polymorphisms (SNPs) correlated with recurrence-free and progression-free survival in NMIBC. They found that lead SNP rs12885353 on chromosome 14 was associated with an increased expression of SCFD1 and associated with recurrence-free survival. The heterogeneous cohort was a limitation of this study.

To the best of our knowledge, only two studies have used AI to analyze genetic markers. Frantzi et al. [91] used an SVM to detect primary and recurrent tumors using 106 peptide genome markers. The study included intermediate- and high-risk NMIBC patients, and achieved a sensitivity and specificity of 0.88 and 0.51, respectively. The author suggested that higher performance could be obtained when combined with cytology, yielding 100% sensitivity. Unfortunately, there was not enough data to show a correlation between the clinical and genetic markers. In a study by Bartsch et al. [92], genetic programming (GP) was used to discover mathematical models to predict recurrence over 5 years of follow-up based on using the whole genome profiling of the bladder tumor specimens. The highest performance was achieved with a three-gene rule which predicted recurrence with a specificity of 0.71 and a specificity of 0.67 in a test set. Both studies were limited by the lack of an external dataset for validation.

In summary, based on the data collected on Table 4, the TERT and FGFR3 genomes were the most widely tested markers among the included studies. They were evaluated in six studies ([69,70,71,72,73,74]) and associated with accuracies ranging from 0.877 to 0.933 and a sensitivity ranging from 0.57 to 100%. The best results were found by Batista et al. [73] using the Uromonitor urine-based test which included both TERT and FGFR3 genomic markers combined with cytology. Notably, the sensitivity of any test including FGFR3 is higher in higher-grade tumors, unlike TERT. Studies assessing the Xpert urine-based test [76,77] found an accuracy of 0.79 and a sensitivity of 0.80. Other tests discussed included Cxbladder [75], EpiCheck [81], and ADXBLADDER [82]. The highest accuracy of 0.856 was found with EpiCheck [75], while the best sensitivity of 0.93 was found for Cxbladder [75]. The three tests had a higher sensitivity for high-grade tumors. The UroVysion test evaluated in two studies [45,80] predicted recurrence in patients undergoing intervesical BCG treatment with an accuracy ranging from 0.703 to 0.77 and a sensitivity from 0.5 to 0.59. Studies evaluating the genetic markers such as NMP22 [83] and DNA methylation [84,85,86] have good results with a sensitivity ranging from 0.615 to 0.94, the highest found in [86] for ZNF154 methylation. Eight statistical studies [70,74,78,79,87,88,89,90] found a correlation of multiple genetic markers with recurrence but did not evaluate test performance characteristics. Some of the markers were strongly correlated with the tumor stage and grade, such as Circ-ZKSCAN1 [78]. Maldonado et al. [87] correlated multiple markers with recurrence in low-grade T0 NMIBC. A study by Lian et al. [79] also showed a correlation between the high-tumor grade and gender with their markers in predicting recurrence. A study correlating HLA genotypes [89] with recurrence rates, while two studies [70,90] suggested the use of SNPs for predicting tumor recurrence. Two AI algorithms were developed genetic markers, an SVM and rule-based ensemble algorithms developed using genetic programming (GP) [91,92]. The SVM had a higher sensitivity of 0.88, however, a three-gene rule constructed using GP had a higher specificity of 0.69. There were high NPVs for the majority of these tests, indicating that they minimize the use of cystoscopy for follow-up. However, a high false positive rate for some of the tests could lead to unnecessary cystoscopy and biopsy. Most of the studies, however, need validation to widely mandate their clinical use. Adequate prospective studies with a long follow-up are required to confirm the impact of these tests on disease management. In the following Table 5, we explore studies using combined classes of markers.

## 6. Combined Markers

Recently, efforts have been directed towards using combinations of markers from different classes to enhance the prediction of MIBC recurrence and progression. These are summarized in Table 5. Only one study combined radiological data with clinical data (Xu et al. [93]). Radiomic features were extracted from multiparametric MRI images using an SVM, achieving an accuracy of 0.755. A combination of radiomic and statistically selected clinical markers yielded an accuracy of 0.809 and a better accuracy for the prediction of recurrence over 2 years. The use of a small number of clinical markers limited this study. The author suggested that genomic markers could be used to increase the overall performance.

More than half of the combined studies used clinical and pathological markers in their models, and many of these studies used machine learning as their core methodology. Starting with Borgi et al. [94], they developed a classifier based on association rules (CBA) with 24 attributes. The most common markers were age, gender, smoking history, and tumor-related attributes (such as stage and of multiplicity). Their model achieved an accuracy of 0.51 in predicting intravesical recurrence in patients who received BCG. The results are limited due to the retrospective nature of the study, heterogeneous and incomplete data, and imbalanced classes between no-recurrence and recurrence cases. SVM models were used by many authors. Lee and other colleagues [95], for example, show that the presence of intravesical prostate protusion (IPP) and other clinicopathological markers in an SVM model yielded accuracies of 0.754 to 0.803. Despite their promising results, further studies are necessary to confirm the utility of IPP as a predictor. Hasnain et al. [96] used an SVM for the prediction of NMIBC and MIBC recurrence at 1, 3, and 5 years using 52 pathological, radiological, and clinical markers. They found the superiority of a 1 year predictive model vs. 3 and 5 years, using a metaclassifier algorithm consisting of SVM, bagged SVM, KNN, AdaBoost, RF, and gradient-boosted trees for NMIBC and MIBC. Regarding deep learning, Lucas and Jobczyk [97,98] used such a model for recurrence prediction. Lucas constructed two models in their study [97] that predicted recurrence within 1 and 5 years using 204 and 200 histopathological markers extracted from histopathological slides with four and three clinical markers including any previous malignancies, tumor stage (Ta vs. other), intravesical chemotherapy, and smoking history, respectively. The best accuracy was obtained from a combined 1-year recurrence prediction model. Hence, the author concluded that markers extracted from digital histopathological images combined with other markers could be useful for recurrence prediction. Jobczyk et al. [98] used Cox proportional-hazards (CPH) deep neural networks to predict recurrence for up to 10 years. The study combined both EORTC and CUETO scores with other markers including gender, age, and the type of intravesical treatment. The main performance metric for the model was the C-index, derived by scoring different results relative to the type of treatment. They found a different C-index for chemotherapy (0.666) and TURBT and BCG immunotherapy (0.651). Despite the large cohort of this study, the author is discrete in using their model in high-risk patients that did not receive any intravesical therapy. EORTC and CUETO scores were also used by Vedder et al. [99] and validated in different cohorts to predict the NMIBC recurrence, however, without using AI. Vedder used statistical methodology, finding a C-index ranging from 0.55 to 0.61, suggesting the superiority of ML in prediction. Getzler et al. [100] found an increased performance with the addition of the EORTC score to NLR. Cambier and colleagues [101] studied intervesical BCG recurrence within 1–5 years with nomograms and found a prior recurrence rate and number of tumors as significant prognostic factors for recurrence after using EORTC to stratify patients into intermediate- and high-risk categories. They achieved a C-index of 0.56 and found the highest recurrence rate in T1G3 patients. However, the high recurrence risk may be influenced by the lack of re-TURBT and exclusion of CIS patients. Kim et al. [102] studied Korean patients using a nomogram with similar markers to those used in Jobcyzk [98], although excluding age and gender while including gross hematuria and previous or concomitant upper urinary tract cancer in their analysis. The nomogram resulted in an almost identical C-index despite there being insufficient data for stratifying models according to each intervesical treatment as Jobcykz did. The same markers were examined by Ali-El-Dien et al. [103] with the addition tumor stage and intervesical therapy. They also used nomograms, finding a C-index of 0.694 in predicting 5-year recurrence. However, a low number of BCG-treated cases limited the validity of the results. Evaluating patients with multiple low-grade-T0 tumors, Nerli’s study [104] correlated the use of tobacco and absence of intravesical BCG as significant predictors of tumor recurrence. It should be noted that many studies neglected low-grade categories, making this study a unique. Another unique study by Zgao et al. [105] evaluated the controlling nutritional status (CONUT) score (a score designed to screen for undernutrition), using a cut-off score of 1 and other markers such as age, smoking history, as well as tumor stage and grade. They found a sensitivity of 0.8486 and a C-index of 0.851 using a nomogram model. Notably, this was the best C-score among the aforementioned studies, although the study was comprised of a small number of subjects and was retrospective. An additional two studies included surgical parameters. Suarez-Ibarrola et al. [106] used a surgical checklist with eight elements that should be used in a high-quality TURBT. Two of the elements were significant predictors for recurrence over 3 years, including the number and location of the tumors. Li et al. [107] evaluated different operative methods as predictors of the decreased recurrence rate. They concluded that pin-ERBT (a method using a pin-shaped electrode for tumor resection) was the most effective procedure for reducing recurrence. In addition to the operative method, age, smoking, and tumor grade were statistically correlated with recurrence. A notable drawback of this study is that multiple larger tumors (more than 3 cm) could not be completely resected using pin-ERBT, and few samples had such characteristics.

Four combined studies [108,109,110,111] used genomic markers. Ajili et al. [108] used an ANN with clinicopathological markers and a single genomic marker (CD34 gene) to predict the intravesical recurrence. The model had an accuracy of 0.957. The model was trained on a small dataset, and validation using a larger external dataset would be needed to confirm the utility of this model. Two additional studies [109,110] evaluated the use of protein panels for recurrence prediction. Zhan et al. [109] used MALAT1, PCAT-1, and SPRY4-IT1 biomarkers, and found a sensitivity of 0.625. Additionally, the tumor stage was statistically correlated as a predictor and PCAT-1 was found to be an independent predictor. Gogalic et al. [110] used common clinicopathological markers with ECadh, IL8, MMP9, EN2, and VEGF biomarkers. A model using a combination of these markers achieved an AUC of 0.84. Finally, Lopez et al. [111] used 171,295 SNP to examine the role of those markers in predicting recurrence. Despite the prospective, detailed data and long follow-up, the author did not find any correlation of SNP in recurrence predictability.

As discussed in Table 5, many authors used combined markers and AI to predict tumor recurrence. The most used AI algorithm was SVM, which was used in three studies [93,95,96] with radiomic, clinical, and pathological markers, achieving an overall accuracy of approximately 0.75 and a sensitivity between 0.593 and 0.774. Deep learning algorithms were used in three studies [96,97,98,108] with clinical, pathological, and genomics markers yielding accuracies between 0.65 and 0.975. The classifier based on association rules (CBA) was also used by a single study [94], with an accuracy of 0.51. Statistical methods were used in most studies [99,100,101,102,103,104,105,106,107,109,110,111]. A unique study [98] combined statistical and ML methods, developing a CPH deep neural network. Many of the markers used were consistent among these studies. To illustrate, tumor characteristics such as stage, grade, size, and multiplicity were frequently used. Likewise, intervesical treatment with either BCG or chemotherapy were common clinical markers. Furthermore, the age, gender, smoking history, and previous recurrence were used in more than five studies. Studies using genomic markers still need a further investigation since only four such studies were found [106,108,109,110]. Other studies included unique clinical markers such as CONUT score [105], IPP [95], and NLR [100]. Two studies [106,107] assessed the quality of TURBT as an influence on recurrence risk rates. Common limitations found were the need for more detailed patient history and retrospective design which is inherently vulnerable to bias. Despite the limitations, these studies challenge the validated recurrence prediction systems as EORTC and CEUTO, and have the potential to reduce bias and interobserver variability.

### 6.1. Limitations and Strengths

Despite the adequate number of studies that show promising results in predicting NMIBC recurrence, major limitations were found in data that were retrospective, nonhomogeneous, the lack of interobservers’ consistency and small number to validate such studies. Additionally, many of them only used statistical methods, treating the problem of prediction as one of linear regression. However, AI techniques can successfully solve both linear and non-linear problems as well as both classification and regression. Hence, AI models are more robust and well-generalized with the potential to predict new unseen cases, revealing that AI strength can assist in developing more accurate personalized management systems that are objective and diminish any biases due to subjectivity.

### 6.2. Conclusion and Future Trends

NMIBC recurrences are common in BC, with recurrence rates ranging from 70% to 80%. In this survey, we covered several techniques as radiomics, histopathological, clinical, genomics, and any other combinations of them that can predict NMIBC recurrence and help manage and individualize management of this disease. From approximately 70 studies, our conclusions show initial results for radiomics using intensity markers in MRI or CT images that had an accuracy range of 0.886–0.926 using statistical methods and not AI. Histopathological markers yielded accuracies ranging from 0.72 to 0.90 using AI approaches. Common histopathological markers used were tumor multiplicity, tumor size, tumor grade, and tumor stage. Furthermore, an AI model using an NLR had a range of accuracies from 0.638 to 0.923. This marker was used in several studies using clinical markers and other studies using additional markers. Commonly used genetic markers were TERT and FGFR3 with accuracies ranging from 0.877 to 0.9233. In addition, many genomic studies used urine and serum tests such as Cxbladder, EpiCheck, Xpert monitor, and many others to enhance the prediction of recurrence. Studies using combinations of markers from different classes achieved high accuracies in predicting tumor recurrence. Most of the AI-based studies used SVM. Other AI techniques were used to analyze the combinations of markers. The future of disease management in NMIBC and many other diseases will use AI-based models to reduce bias and interobserver variability, but this will require wide-scale effort to develop the large, high-quality datasets needed to train such models. Despite the increased power of AI and its added value in the personalized medicine field assessing recurrence risk, its ability to predict recurrences with such medical markers cannot yet be treated as a gold standard. Our review investigated the usage of AI-models regardless of their possibility to fail, so intense studies should be investigated to assure its benefit.

## Figures and Tables

**Table 1:** Literature review on the use of radiomic markers to predict recurrence in NMIBC.

Study	Study Aim	Markers	AI Model	Results	Findings
Wang, H. et al. [46]	Current recurrence prediction after TURBT or cystectomy from MRI images—**11 patients.**	**Radiomics:** Intensity marker from DCE and DWI images: high intensities on DWI and DCE ROIs while low intensities on ADC map.	**Radiomics:** Statistical analysis only. (K- statistical method for the consistency of uroradiologists results.)	**Radiomics:** DWI: Acc: 0.926. Sen: 1.00. Spe: 0.818. DCE: Acc: 0.593. Sen: 0.813. Spe: 0.273.	DWI-MRI has a better accuracy for predicting current recurrence than DCE.
El-Assmy, A. et al. [47]	Current recurrence prediction after TURBT from MRI images—**47 patients.**	**Radiomics:** Intensity marker from DWI images.	**Radiomics:** Statistical analysis only. (K-statistical method for the consistency of uroradiologists’ results.)	**Radiomics:** Acc: 0.915. Sen: 0.916. Spe: 0.913.	DWI-MRI can be used to predict current recurrences with good accuracy.
Yang, Z. et al. [48]	Current recurrence prediction using FDG ^1^ PET/CT images—**35 patients.**	**Radiomics:** An intensity marker: standardized uptake value (SUV) from FDG PET/CT and additional oral hydration–voiding–refilling pelvic images.	**Radiomics:** Experienced nuclear medicine physicians with statistical analysis only.	**Radiomics:** Acc: 0.886. Sen: 0.917. Spe: 0.87.	FDG PET/CT with additional pelvic images can be used to predict recurrence presence.
Alongi, P. et al. [15]	Current recurrence prediction from FDG ^1^ PET/CT images—**41 patients.**	**Radiomics:** An intensity marker: standardized uptake value (SUV) from FDG PET/CT.	**Radiomics:** Experienced nuclear medicine physicians with statistical analysis only: Bayes’ law.	**Radiomics:** Acc: 0.90. Sen: 0.87. Spe: 0.94.	FDG PET/CT can effectively predict current recurrences.

^1^ Fluorine-18 fluorodeoxyglucose.

**Table 2:** Literature review on using histopathological parameters and IHC markers to predict recurrence in NMIBC.

Study	Study Aim	Markers	AI Model	Results	Findings
Chen, J.-X. et al. [49]	Predicting recurrence after TURBT followed by intervesical therapy from histopathological parameters and IHC markers—**72 patients.**	**Histopathological slides:** 2 markers including: Ki67 labeling index (LI) and VEGF scoring.	Statistical analysis only (Cox regression analysis).	*p* < 0.05 considered to be significant. Highest recurrence was for cut-off values of: Ki67 LI > 25%, VEGF scoring > 8.	Grading with Ki67 Li with VEGF scoring can predict recurrence.
Li, G. et al. [50]	Predicting recurrence within 5 years for T1 NMIBC after TURBT from histopathological markers—**426 patients.**	**Histopathological slides:** 4 markers including: tumor multiplicity, tumor size, tumor grade, tumor stage, and the presence of squamous differentiation.	Statistical analysis only (Cox regression analysis).	*p* < 0.05 considered to be significant.	Squamous differentiation was an independent predictor of recurrence with poor response to intravesical therapy.
Xu, H. et al. [51]	Predicting recurrence after TURBT from histopathological markers—**869 patients.**	**Histopathological slides:** 5 markers including: tumor multiplicity, tumor size, tumor grade, tumor stage, and the presence of squamous and/or glandular differentiation	Statistical analysis only (Cox regression analysis).	*p* < 0.05 considered to be significant.	Squamous and/or glandular differentiation was/were an independent prognostic predictor of recurrence.
Zhao, G. et al. [52]	Prediction of recurrence from histopathological slides for T1 NMIBC—**248 patients.**	**Histopathological slides:** 2 markers including: UCGD ^1^ and LVI ^2^.	Statistical analysis (Cox regression models).	*p* < 0.05 considered to be significant.	Glandular differentiation and LVI could be used as predictors for recurrence.
Chamie, K. et al. [53]	Prediction of recurrence rates in 2, 5, and 10 y for high-grade NMIBC patients—**7410 patients.**	**Pathological:** A single significant marker: tumor stage.	Statistical analysis only. (Fine-gray competing-risks regression.)	Recurrence cumulative incidence rates for: **2-years:** 0.611. **5-years:** 0.695. **10-years:** 0.743.	Tumor stage was the only significant predictor correlated with higher rates of recurrence.
Urdal, J. et al. [54]	Predicting recurrence from histopathological slides—**42 patients.**	**Histopathological slides:** 6 textural markers.	Local binary pattern (LBP) and local variance (VAR) operators followed by a RUSBoost classifier.	***Validating:*** **Histopa-thological slides:** Acc: 0.72. Sen: 0.843. Spe: 0.446.	Textural analyses for histopathological slides promises a good predictability for recurrence.
Tokuyama et al. [44]	Predicting recurrence after TURBT in 2 years from histopathological slides—**125 patients.**	**Histopathological slides:** 79 including nuclear morphological and textural markers.	SVM	***Testing:*** **Histopa-thological slides:** Acc: 0.90. Sen: 0.80. Spe: 1.00.	The SVM model using pathological markers only has a good potential to predict recurrence.

^1^ Urothelial carcinoma with glandular differentiation; ^2^ lymphovascular invasion.

**Table 3:** Literature review on the use of clinical markers to predict recurrence in NMIBC.

Study	Study Aim	Markers	AI Model	Results	Findings
Mano, R. et al., [55]	Predicting recurrence from clinical markers—**107 patients.**	**Clinical:** A single significant marker, namely NLR ^1^.	Statistical analysis only (Cox regression models and standardized cut-off-finder algorithm).	*p* < 0.05 considered to be significant. Best Cut-off value was at NLR > 2.43.	NLR > 2.43 has the potential to precisely predict recurrence.
Rubinstein, J. et al. [56]	Prediction of recurrence in T1HG patients in 1 year using clinical markers from 2 cohorts—**73 patients.**	**Clinical:** 2 markers: age and NLR.	**Clinical:** decision tree.	***Testing and/or validating:*** not reported. ***Training:*** **Clinical:** Cohort 1: Acc: 0.923. Sen: 0.100. Spe: 0.80. Cohort 2: Acc: 0.638. Sen: 0.75. Spe: 0.56. Combined: Acc: 0.739. Sen: 0.86. Spe: 0.62.	NLR > 2.5 decision node shows good prediction for recurrence, and cohort 1 has the highest prediction performance.
Albayrak, S. et al. [57]	Predicting recurrence from clinical markers—**86 patients.**	**Clinical:** 2 significant markers, including: NLR and age.	Statistical analysis only. (Multiple linear regression model.)	*p* < 0.05 considered to be significant.	NLR and age considered to be significant predictors for recurrence.
Ferro, M. et al. [58]	Predicting recurrence from clinical inflammatory markers for high-grade T1 NMIBC treated with BCG—**1382 patients.**	**Clinical:** 3 significant markers including NLR, ESR ^2^, and mGPS 1 ^3^.	Statistical analysis only (Cox regression models).	*p* < 0.05 considered to be significant.	NLR and ESR inflammatory markers could predict recurrence, while mGPS 1 can increase the risk of recurrence.
Srougi, V. et al. [59]	Detection and prediction of recurrence in the first 3 years from urinary biomarkers panel—**134 patients.**	**Urinary:** 2-biomarker panel with PAI-1 and IL-8.	**Urinary:** Cut-off values at: PAI-1 < −0.266 and IL-8 < 0.047.	**Urinary:** Acc: 0.793. Sen: 0.868. Spe: 0.381.	Urinary markers panel can predict recurrence with a poor specificity.
Rosser et al. [60]	Prediction of the presence of recurrence from urinary markers—**125 patients.**	**Urinary:** 10 individual models using the following biomarkers—IL8, MMP9, MMP10, SERPINA1, VEGFA, ANG, CA9, APOE, SERPINE1, and SDC1. **Combined**: A single model that integrates the aforementioned biomarkers.	The 11 models used nonparametric ROC analyses.	***Validating:*** Best 2 models were: **Combined:** Acc: 0.84. Sen: 0.79. Spe: 0.88. AUC: 0.904. **SER-PINA1 model:** Acc: 0.78. Sen: 0.87. Spe: 0.72. AUC: 0.864.	The combination of the 10 biomarkers outperformed any single biomarker. Urinary **SERPINA1** provided the highest predictive performance among other biomarkers.
Chevalier, M.F. et al. [61]	Prediction of recurrence after BCG from urinary biomarkers—**28 patients.**	**Urinary:** 2 markers, T cells and monocytic myeloid-derived suppressor cells (M-MDSCs).	**Urinary:** linear regression with statistical analysis.	**Urinary:** *p* < 0.05 considered to be significant. best cut-off was at (T cell / M-MDSC) < 1 with *p* < 0.0001.	A ratio of T-cell to M-MDSC less than 1 shows an increased risk of recurrence.
Alberice, J.V. et al. [62]	Prediction of recurrence from urinary biomarkers—**48 patients.**	**Urinary:** 5 metabolite markers including Nε, Nε, Nε-trimethyllysine, N-acetyltryptophan, dopaquinone, leucine, and hypoxanthine.	**Urinary:** Statistical analysis only from liquid chromatography (LC-MS) and capillary electrophoresis–mass spectrometry (CE-MS).	**Urinary:** *p* < 0.05 considered to be significant.	**Urinary:** Metabolite markers can be used to predict recurrence, where higher concentrations of dopaquinone, leucine, and hypoxanthine correlate with an increased recurrence in low risk patients, while Nε, Nε, Nε-trimethyllysine, and N-acetyltryptophan correlate with an increased recurrence in high-risk patients.
Naselli, A. et al. [63]	Predicting recurrence for 1 year after different operative methods: NBI-TUR ^4^ and WL-TUR ^5^—**148 patients.**	**Clinical:** A significant marker—operative methods.	Statistical analysis only. (Logistic regression.)	(*p* < 0.05) considered to be significant. Recurrence rates were: **NBI-TUR:** 0.329. **WL-TUR:** 0.514.	NBI modality decreases the 1 y-recurrence risk more than WL.
Sfakianos, J.P. et al. [64]	Predicting recurrence over 5 years from restaging TURBT before BCG therapy for high-grade NMIBC. **1021 patients.**	**Clinical:** Number of TURBT (single/multiple).	Statistical analysis. (logistic regressions.)	(*p* < 0.05) considered to be significant. Recurrence rates were: **Single TUR:** 0.772. **Restaging TUR:** 0.616.	Restaging TURBT can decrease the rate of recurrence for HG NMIBC patients.
Culpan, M. et al. [65]	Prediction of recurrence after cystoscopy delay—**407 patients.**	**Clinical:** 2 significant markers—the number of recurrences and the cystoscopy delay time (62–147 days) and (>147 days).	Statistical analysis. (Multivariable logistic regression model.)	*p* < 0.05 considered to be significant.	Delay of 2–5 months in follow-up cystoscopy increases the risk of recurrence.
Lu, J. et al. [66]	Prediction of recurrence undergoing different intravesical therapies—**12,464 patients.**	**Clinical:** A single marker—type of intravesical therapy.	Statistical analysis: Network meta-analysis based on a Bayesian random-effects model.	Top 3 treatment based on AUC ^6^: **GEM:** 0.92. **BCG:** 0.82. **IFN:** 0.78.	GEM, BCG, and IFN are the top three effective drugs to decrease recurrence.
Uhlig, A. et al. [67]	Predicting recurrence from gender clinical marker after BCG treatment—**23,754 patients.**	**Clinical:** A single marker—gender.	Statistical analysis only. (Random effect meta-analysis.)	(*p* < 0.05) considered to be significant.	Women are more likely to have higher risks of recurrence than males and a correlation between impaired BCG and female patients was found.
Van Osch, F.H.M. et al. [68]	Prediction of recurrence from a clinical marker—**722 patients.**	**Clinical:** A single marker—smoking history.	**Clinical:** Statistical analysis only (Cox regression models).	**Clinical:** *p* < 0.05 considered to be significant. Smoking cessation was *p* = 0.352.	The study shows poor association between smoking cessation and recurrence due to the small number of patients that quit smoking.

^1^ Neutrophil-to-lymphocyte ratio; ^2^ erythrocyte sedimentation rate; ^3^ mGPS score 1 for patients with an elevated CRP (>10 mg/L); ^4^ narrow-band imaging transurethral resection; ^5^ white-light transurethral resection; ^6^ the AUC used is SUCRA—the surface under the cumulative ranking curve, used for the overall ranking in each treatment.

**Table 4:** Literature review on genomics biomarkers to predict NMIBC recurrence.

Study	Study Aim	Markers	AI Model	Results	Findings
Kinde, I. et al. [69]	Prediction of recurrence from DNA genomics mutation markers using 2 cohorts—**90 patients**.	**Genomics:** A single marker—TERT mutation.	**Genomics:** Statistical analysis with testing laboratories.	**Genomics:** For the follow-up cohort: Acc: 0.933. Sen: 1.00. Spe: 0.857.	TERT biomarker is a significant predictor for recurrence.
Rachakonda, P.S. et al. [70]	Predicting recurrence after TURBT from DNA genomic markers—**327 patients.**	**Genomics:** 2 significant markers—TERT gene mutation and rs2853669 polymorphism.	Statistical analysis (Cox model).	*p* < 0.05 considered to be significant.	TERT mutation within rs2853669 polymorphism can be used as predictors for recurrence.
Beukers, W. et al. [71]	Prediction of recurrence within 1 year from genomics biomarkers—**977 patients.**	**Genomics:** 3 markers including FGFR3, TERT, and OTX1 genes.	Testing laboratories.	**LG recurrence:** Sen: 0.57. Spe: 0.59. **HG recurrence:** Sen: 0.72.	Although the combination of the 3 genes shows low sensitivity in LG recurrences, it has been noticed that those markers can better predict HG recurrence.
Kandimalla, R. et al. [72]	Prediction of recurrence from genomics biomarkers—**196 patients.**	**Genomics:** 4 biomarkers including a methylation assay of OTX1, ONECUT2, and OSR1 combined together with FGFR3.	**Genomics:** Logistic regression with a cut-off value at 0.58 for assay.	***Validating:*** **Genomics:** Sen: 0.79. Spe: 0.90 AUC: 0.886.	The combined methylation assay with FGFR3 can improve the accuracy of predicting recurrence.
Batista, R. et al. [73]	Prediction presence of recurrence from genomics mutation markers—**185 patients**.	**Genomics:** 2 markers—TERT and FGFR3 mutations.	**Genomics:** Uromonitor^®^ testing for detection mutations.	**Genomics:** For the follow-up cohort: Uromonitor^®^: Acc: 0.877. Sen: 0.735. Spe: 0.932. Cystoscopy: Acc: 0.893. Sen: 0.794. Spe: 0.932. Combined: Acc: 0.902. Sen: 1.00. Spe: 0.864.	Uromonitor^®^ test shows similar results comparing to cystoscopy. A combination of both markers can be used to achieve a high predicting recurrence performance.
Park, J. et al. [74]	Prediction of recurrence after BCG treatment for T1G3 NMIBC from genomics biomarkers—**61 patients.**	**Genomics:** 7 biomarkers—p53, pRb, PTEN, Ki-67, p27, FGFR3, and CD9.	Statistical analysis only (Cox regression).	*p* < 0.05 considered to be significant.	No significant results were shown for predicting recurrence for T1G3 NMIBC using the corresponding markers.
Kavalieris, L. et al. [75]	Prediction of recurrence presence from mRNA genomics biomarkers—**763 patients.**	**Genomics:** 5 biomarkers including IGFBP5, HOXA13, MDK, CDK1, and CXCR2 mRNA genes.	**Genomics:** Cxbladder test with logistic regression.	***Validating:*** **Genomics:** Sen: 0.93. Spe: 0.34.	Cxbladder test has the potential to rule out recurrence cases due to high NPV.
F. Johannes P. van Valenberg et al. [76]	Prediction of recurrence from mRNA genomics biomarkers—**239 patients.**	**Genomics:** 5 mRNA biomarkers including ABL1, ANXA10, UPK1B, CRH, and IGF2.	**Genomics:** Xpert Monitor test with linear regression models.	***Testing:*** **Genomics:** Acc: 0.79. Sen: 0.74. Spe: 0.80.	Comparing to UroVysion and cystoscopy in the same study, Xpert Monitor has the highest sensitivity and NPV values compared to the similar accuracies for Xpert and cystoscopy.
Elsawy, A.A. et al. [77]	Prediction of recurrence from mRNA genomics biomarkers—**181 patients.**	**Genomics:** 5 mRNA biomarkers including ABL1, ANXA10, UPK1B, CRH, and IGF2.	**Genomics:** Xpert Monitor test with statistical analysis (Cox regression.)	***Validating:*** **Genomics:** Sen: 0.737. Spe: 0.796.	Xpert Monitor shows a high association with early recurrence in addition to its good predictability.
Bi, J. et al. [78]	Prediction of recurrence from the Circ-RNA genomics biomarker—**68 patients.**	**Genomics:** A single biomarker—Circ-ZKSCAN1.	Statistical analysis only.	*p* < 0.05 considered to be significant.	High expressions of Circ-ZKSCAN1 show a high correlation with decreasing recurrence. Furthermore, the marker has strong correlation with tumor stage and grade.
Lian, P. et al. [79]	Prediction of recurrence from lncRNA genomics markers—**343 patients**.	**Genomics:** 8 significant markers including APCDD1L-AS1, FAM225B, LINC00626, LINC00958, LOC100996694, LOC441601, LOC101928111, and ZSWIM8- AS1.	**Genomics:** statistical analysis only (Cox regression).	*p* < 0.05 considered to be significant.	The eight lncRNA genes have the potential to predict recurrence.
Liem, E.I.M.L. et al. [45]	Prediction of recurrence within 3 months from DNA markers after intravesical treatment for intermediate- and high-grade NMIBC. **114 patients**.	**Genomics:** A single marker—FISH.	**Genomics:** UroVysion testing laboratories. and statistical analysis only (Cox regression).	**Genomics:** Week 0, Before BCG: Acc: 0.54. Sen: 0.44. Spe: 0.59. Week 6, after TURB: Acc: 0.67. Sen: 0.21. Spe: 0.88. 3 months after TURB: Acc: 0.77. Sen: 0.59. Spe: 0.84.	A positive FISH test at 3 months after TURB had a 4.6 times greater risk of tumor recurrence than a negative FISH test. Furthermore, the significant correlation of recurrence was only noticed after 3 months post-TURBT.
Kojima, T. et al. [80]	Prediction presence of recurrence within 3 months from DNA markers after intravesical treatment—**468 patients**.	**Genomics:** 2 individual tests using the following markers—UroVysion test and urine cytology. **Combined:** A combination of two consequence UroVysion tests.	**Genomics:** testing laboratories. **Combined:** Statistical analysis only. (Kappa coefficient.)	**Genomics:** UroVysion test: Acc: 0.703. Sen: 0.50. Spe: 0.724. Urine cytology: Acc: 0.908. Sen: 0.045. Spe: 0.998. **Combined:** Recurrence rates for: Both positive: 0.148. Either positive: 0.072. Both negative: 0.01.	**Genomics:** Although urine cytology shows the highest accuracy, the author concludes that the UroVysion test has the best sensitivity to better predict recurrence than urine cytology. **Combined:** A use of two consecutive UroVysion tests promises a good predictability of recurrence.
Witjes, J.A. et al. [81]	Prediction of recurrence from DNA methylation genomics markers—**353 patients**.	**Genomics:** 15 DNA methylation biomarkers.	**Genomics:** Bladder EpiCheck (BE) testing laboratories, a cut-off EpiCheck score > = 60 is considered a positive result.	**Genomics:** For entire cohort: Acc: 0.856. Sen: 0.682. Spe: 0.88. AUC: 0.82. Without LG Ta recurrences: Acc: 0.883. Sen: 0.917. Spe: 0.88. AUC: 0.94.	EpiCheck test can more effectively predict the recurrence for high-grade NMIBC.
Roupret, M. et al. [82]	Prediction presence of recurrence from a DNA genomics marker after TURBT—**127 patients**.	**Genomics:** A single marker—MCM5.	**Genomics:** ADXBLADDER testing laboratories.	**Genomics:** Acc: 0.688. Sen: 0.449. Spe: 0.711. AUC: 0.57.	ADXBLADDER test has the potential to detect the MCM5 marker that can help in predicting recurrence.
Önal, B. et al. [83]	Prediction of the presence of recurrence from genomics biomarkers—**65 patients.**	**Genomics:** A single marker—NMP22.	NMP22 cut-off value of 6.4.	**Genomics:** Sen: 0.854. Spe: 0.765.	Compared to cytology, the NMP22 immunoassay genomic marker showed the highest results for LG patients.
Su, S.-F. et al. [84]	Prediction of recurrence from DNA methylation genomics biomarkers—**90 patients.**	**Genomics:** 3 markers including SOX1, IRAK3, and L1-MET genes.	**Genomics:** Feature selection: Logistic regression. Classification: Recurrence risk score cut-off value of > 0.	***Testing:*** **Genomics:** Sen: 0.86. Spe: 0.80. AUC: 0.90.	The hypermethylation of SOX1, and IRAK3 as well as hypomethylation of L1-MET genes can improve the predictability of recurrence.
Shindo, T. et al. [85]	Prediction of current and late recurrence from mRNA genomics biomarkers—**132 patients.**	**Genomics:** Methylation of 4 markers including miR-9-3, miR-124-2, miR-124-3, and miR-137 genes.	**Genomics:** Current recurrence: Cut-off values at: miRNA methylation score (M - Score) >= 3, miR-137 > 5.2%; miR-124-2 > 5.2%; miR-124-3 > 12.0%; and miR-9-3 > 7.2%. Late recurrence: Statistical analysis only (Cox regressions).	**Current recurrence:** Sen: 0.615. Spe: 0.74. AUC: 0.71. **Late recurrence:***p* < 0.05 considered to be significant.	M-score is a significant predictor for current and late recurrences, and high levels of mRNA methylation increase the risk of recurrence.
Reinert, T. et al. [86]	Prediction of recurrence within 1 year from DNA methylation markers—**184 patients.**	**Genomics:** 5 markers including HOXA9, POU4F2, TWIST1, VIM, and ZNF154.	**Genomics:** Cut-off values at: HOXA9 =0.077, POU4F2 = 0.371, TWIST1 = 0.405, VIM = 0.368 and ZNF154 = 1.51. Statistical analysis (Cox regression).	**Genomics:** HOXA9: Acc: 0.855. Sen: 0.93. Spe: 0.55. AUC: 0.78 POU4F2: Acc: 0.822. Sen: 0.88. Spe: 0.64. AUC: 0.80. TWIST1: Acc: 0.802. Sen: 0.90. Spe: 0.43. AUC: 0.76. VIM: Acc: 0.835. Sen: 0.90. Spe: 0.59. AUC: 0.78. ZNF154 Acc: 0.881. Sen: 0.94. Spe: 0.67. AUC: 0.83.	ZNF154 has the highest performance marker for predicting recurrence. Low specificities are also observed.
Maldonado, L. et al. [87]	Prediction of recurrence after TURBT from DNA genomics markers for low grade-T0-NMIBC—**36 patients**.	**Genomics:** 3 significant markers including the promoter methylation of CCND2, CCNA1, and CALCA genes.	**Genomics:** PCR testing laboratories.	*p* < 0.05 considered to be significant.	Methylated CCND2, CCNA1, and CALCA genes show significant results for recurrence prediction. Interestingly, CCNA1 is considered to be a suppressor tumor gene.
Bellmunt, J. et al. [88]	Prediction of recurrence within 7.4 years for patients for HGT1 from genomics biomarkers—**62 patients.**	**Genomics:** 9 markers including RHOB, ARID1A, and TP53 mutations, CDKN2A deletion and focal gain in CCNE1, PVRL4, YWHAZ, E2F3-SOX4, and PPARG genes.	Statistical analysis only.	*p* < 0.05 considered to be significant.	The corresponding genomics markers have the potential to predict recurrence in high-grade T1 NMIBC.
Kobayashi, M. et al. [89]	Prediction of recurrence after BCG therapy from human leukocyte antigen (HLA) genetic markers—**195 patients.**	**Genomics:** 2 markers including HLA-B supertypes HLA-B07 and HLA-B44. Zygosity: homozygous or heterozygous.	Statistical analysis (Cox regressions).	*p* < 0.05 considered to be significant.	HLA-B homozygous were more likely than HLA-B heterozygous for intravesical recurrence. Furthermore, B07 and B44 genes decrease recurrence rates. Additionally, combination with CUETO scoring improved the C-index.
Galesloot, T.E. et al. [90]	Predicting recurrence from genomic markers from six cohort—**3400 patients.**	**Genomics:** A single marker— single-nucleotide polymorphisms (SNPs), with 12 SNPs containing 18 candidate tumor genes.	Statistical analysis: GWASs ^1^ with Cox model.	SNPs with *p* < 5 × 10${}^{-5}$ considered to be significant. **Genomics:** Highest p-value for: SNP: SNP (rs12885353) locus. Candidate gene: SCFD1 ^2^ tumor gene.	The SNP rs12885353 was the most associated locus with recurrence. Additionally, SCFD1 ^2^ was the most associated gene with a decreased risk of recurrence.
Frantzi, M. et al. [91]	Prediction of recurrence presence from peptide genomics biomarkers—**636 patients.**	**Genomics:** 106 peptide biomarkers majority for collagen fragments and Apolipo protein (Apo A-I) peptide sequences.	**Genomics:** SVM.	***Validating:*** **Genomics:** Sen: 0.88. Spe: 0.51. AUC: 0.75.	The use of urine-based peptide biomarkers with ML promises a good predictability for recurrence.
Bartsch, G. et al. [92]	Prediction of recurrence within 5 years after TURBT from genomics biomarkers—**100 patients.**	**Genomics:** 21 biomarkers key-genes.	**Genomics:** Genomic programming (GP)—rule-based ensemble classifiers.	***testing:*** **Genomics:** 5-gene combin- ation rule: Sen: 0.69. Spe: 0.62. 3-gene singular rule: Sen: 0.71. Spe: 0.69.	Genomic programming can help build a good model for predicting recurrence. Additionally, both rules show similar results.

^1^ Genome-wide association studies. ^2^ (Sec1 family domain-containing protein 1) on chromosome 14, also known as SLY1 and is found on SNP(rs12885353) locus.

**Table 5:** Literature review on using combined markers to predict recurrence in NMIBC.

Study	Study Aim	Markers	AI Model	Results	Findings
Xu, X. et al. [93]	Prediction of recurrence in the first 2 years from radiomics and clinical markers—**71 patients.**	**Radiomics:** 32 textural markers. **Clinical:** A single marker: Muscle-invasive status (MIS). **Combined:** 33 markers (radiomics + clinical).	**Radiomics:** SVM-RFE ^1^. **Clinical:** Statistical analysis only. **Combined:** Recurrence risk threshold of 0.55.	***Validation:*** **Radiomics:** Acc: 0.755. Sen: 0.777. Spe: 0.738. AUC: 0.821. **Clinical:** *p* < 0.05. **Combined:** Acc: 0.809. AUC: 0.838.	The combined model shows the best prediction of recurrence.
Borgi et al. [94]	Predicting recurrence after TURBT followed by BCG treatment—**543 patients.**	**Clinicopathological:** 24 markers including 22 symbolic attributes and 2 continuous attributes.	Classifier based on association rules (CBA).	***Testing:*** **Clinicopathol-ogical:** Acc: 0.51. Sen: 0.688. Spe: 0.385.	The use of association rules exhibits a good sensitivity for recurrence prediction.
Lee, J. et al. [95]	Prediction of recurrence in 5 years from clinicopathological markers after TURBT—**122 patients.**	**Clinicopathological:** Model 1: 10 markers including age, smoking history, cytology results, associated CIS, tumor stage, tumor grade, tumor size, tumor multiplicity, BCG treatment, and prostate volume. Model 2: 11 markers including the above 10 markers with presence of intravesical prostate protusion (IPP) for grade 2–3.	SVM	***Testing and/or validating:*** not reported. ***Training:*** **Clinicopathological:**Model 1: Acc: 0.754. AUC: 0.710. Model 2: Acc: 0.803. AUC: 0.749.	Addition of IPP improved the model predicting recurrence accuracy.
Hasnain, Z. et al. [96] (Study 1)	Prediction of recurrence in the 1^st^ year from clinicopathological markers—**3071 patients.**	**Clinicopathological:** Model 1: A single marker—tumor stage. Model 2: A single marker—tumor stage by TNM 5th edition. Model 3: 52 pathological and clinical markers including the above markers.	**Clinicopathol-ogical:** Model 1: Logistic regression. Model 2: Logistic regression. Model 3: Metaclassifier consisting of SVM, bagged SVM, KNN, AdaBoost, RF, and gradient-boosted trees (GBTs).	***Testing:*** **Clinicopathol-ogical:** Model 1: Sen: 0.826. Spe: 0.593. Model 2: Sen: 0.761. Spe: 0.653. Model 3: Sen: 0.739. Spe: 0.714.	Prediction for first year of recurrence shows best results rather than 3 and 5 years prediction.
Hasnain, Z. et al. [96] (Study 2)	Prediction of recurrence within 3 years from clinicopathological markers—**2955 patients.**	**Clinicopathological:** Model 1: A single marker—tumor stage. Model 2: A single marker—tumor stage by TNM 5th edition. Model 3: 54 pathological and clinical markers including the above markers.	**Clinicopathol-ogical:** Model 1: Logistic regression. Model 2: Logistic regression. Model 3: Metaclassifier consisting of SVM, bagged SVM, KNN, AdaBoost, RF, and gradient-boosted trees (GBT).	***Testing:*** **Clinicopathol-ogical:** Model 1: Sen: 0.774. Spe: 0.631. Model 2: Sen: 0.670. Spe: 0.694. Model 3: Sen: 0.720. Spe: 0.708.	Metaclassifier shows its robustness along predictions of 1, 3, and 5 years of recurrence.
Hasnain, Z. et al. [96] (Study 3)	Prediction of recurrence within 5 years from clinicopathological markers—**2695 patients.**	**Clinicopathological:** Model 1: A single marker—tumor stage. Model 2: A single marker—tumor stage by TNM 5th edition. Model 3: 51 pathological and clinical markers including the above markers.	**Clinicopathol-ogical:** Model 1: Logistic regression. Model 2: Logistic regression. Model 3: Metaclassifier consisting of SVM, bagged SVM, KNN, AdaBoost, RF, and gradient-boosted trees (GBT).	***Testing:*** **Clinicopathol-ogical:** Model 1: Sen: 0.744. Spe: 0.611. Model 2: Sen: 0.619. Spe: 0.698. Model 3: Sen: 0.700. Spe: 0.702.	Although Model 1 shows the highest sensitivity among the 1–2 and 3 years prediction, metaclassifiers maintain the most accuracy and show the best performance.
Lucas, M. et al. [97] (Study 1)	Prediction of recurrence in the 1^st^ year from histopathology slides and clinical markers—**359 patients.**	**Histopathology slides:** 204 markers. **Clinical:** 4 markers including previous malignancies, T stage (Ta vs. other), intravesical chemotherapy, and smoking history. **Combined:** 204 markers (histo + clinical).	**Histopathol-** **ogy slides:** Segmentation: U-net. Feature selection: VGG16. Classification: Bidirectional GRU. **Clinical:** Multivariable logistic regression. **Combined:** Bidirectional GRU.	***Testing:*** **Histopathol-** **ogy slides:** Acc: 0.61. Sen: 0.50. Spe: 0.65. AUC: 0.56. **Clinical:** Acc: 0.69. Sen: 0.10. Spe: 0.94. AUC: 0.58. **Combined:** Acc: 0.65. Sen: 0.30. Spe: 0.80. AUC: 0.62.	The combined histo-clinical markers enhance the model performance especially for 1 y recurrence prediction.
Lucas, M. et al. [97] (Study 2)	Prediction of recurrence in the 5 years from histopathology slides and clinical markers—**281 patients.**	**Histopathology slides:** 200 markers. **Clinical:** 3 markers including: previous malignancies, T stage (Ta vs. other) and intravesical chemotherapy. **Combined:** 203 markers (histo + clinical)	**Histopathol-** **ogy slides:** Segmentation: U-net. Feature selection: VGG16. Classification: bidirectional GRU. **Clinical:** multivariable logistic regression. **Combined:** bidirectional GRU.	***Testing:*** **Histopathol-** **ogy slides:** Acc: 0.67. Sen: 0.93. Spe: 0.38. AUC: 0.72. **Clinical:** Acc: 0.52. Sen: 0.67. Spe: 0.35. AUC: 0.57. **Combined:** Acc: 0.74. Sen: 0.89. Spe: 0.57. AUC: 0.76.	The combined histo-clinical markers demonstrated better recurrence prediction performance using the 5 y model rather than using the 1 y model.
Jobczyk et al. [98]	Prediction of recurrence within 10 years using clinicopathological markers—**3892 patients.**	**Clinicopathological:** 8 markers including gender, age, T stage, tumor grade, No. of tumors, tumor diameter, EORTC and CUETO scores, and type of intravesical treatment.	CPH deep neural network (DeepSurv).	***External Validating:*** **Clinicopathol-ogical:**TURBT and BCG treatment model: C-index: 0.651. mitomycin C treat- ment model: C-index: 0.660.	DeepSurv could be considered to predict recurrence after undergoing various treatments.
Vedder, M.M. et al. [99]	Predicting recurrence for Ta/T1 NMIBC in 10-years from 3 cohorts—**1892 patients.**	**Clinicopathological:** Markers from: The EORTC score: 6 markers: number of tumors, tumor size, prior recurrence rate, T stage, T grade, and concomitant carcinoma in situ. The CUETO score: 7 markers—gender, age, recurrent tumor, number of tumors, T stage, T grade, and concomitant carcinoma in situ.	Statistical analysis (Cox regression based on EORTC and CUETO scores).	**EORTC c-index:** Cohort 1 (Denmark): 0.61. Cohort 2 (The Ne-therlands): 0.55. Cohort 3 (Spain): 0.59. **CUETO c-index:** Cohort 1 (Denmark): 0.56. Cohort 2 (The Ne-therlands): 0.58. Cohort 3 (Spain): 0.59.	The EORTC and CUETO risk scores can predict recurrence.
Getzler, I. et al. [100]	Predicting recurrence from NLR—**113 patients.**	**Clinicopathological:** 2 significant markers including NLR and EORTC score.	Statistical analysis only (Cox regressions).	*p* < 0.05 considered to be significant.	NLR > 2.5 is a significant predictor of recurrence. Additionally, its combination with the EORTC score improves predictability on the whole cohort.
Cambier, S. et al. [101]	Predicting recurrence rates for 1 and 5 years after TURBT followed by 1–3 years BCG treatment—**1812 patients.**	**Clinicopathological:** 2 significant markers including prior recurrence rate and number of tumors.	Statistical analysis only (logistic regression model and nomograms).	***Validating:*** **Clinicopathol-ogical:** AUC: 0.65. C-index: 0.56. Recurrence rates For: 1 year: 0.259. 5 years: 0.413.	Nomogram shows high recurrence rates for high-grade and multiple tumors.
Kim, H.S. et al. [102]	Prediction of recurrence in 5 years after TURBT— **970 patients.**	**Clinicopathological:** 6 markers including gross hematuria, previous or concomitant UTUC ^4^, tumor stage, tumor grade, No. of tumors, and intravesical treatment.	Statistical analysis only (nomograms).	***Internal Validation:*** **Clinicopathol-ogical:** C-index: 0.65.	The first study that shows gross hematuria as a significant predictor for recurrence.
Ali-El-Dein, B. et al. [103]	Prediction of recurrence for 1 year and 5 years—**1019 patients.**	**Clinicopathological:** 4 markers including tumor stage, multiplicity, history of recurrence, and adjuvant intravesical therapy.	Statistical analysis (Cox and logistic regression for nomograms).	**Clinicopathol-ogical:** C-index for: 1 y: 0.649. 5 y: 0.694.	Five-year Nomogram shows a higher predictive performance than one-year Nomogram.
Nerli, R.B. et al. [104]	Predicting recurrence in 5 years of multiple low-grade Ta NMIBC.	**Clinicopathological:** 2 significant markers including use of tobacco and absence of intravesical therapy.	Statistical analysis (Cox models).	All significant markers show *p* < or = 0.001. Recurrence rate: 0.548.	Multiple low-grade Ta NMIBC patients show a higher risks of predicting recurrence.
Zhao, L. et al. [105]	Prediction of recurrence with 1, 3, and 5 years from clinicopathological markers and controlling nutritional status (CONUT ^3^) score—**94 patients.**	**Clinicopathological:** 6 markers including age, history of smoking, pathological T stage, tumor grade, tumor size, and CONUT score.	A nomogram with a cut-off value at CONUT > 1.	***Internal Validating:*** **Clinicopathol-ogical:** Cut-off model: Sen: 0.8485. Spe: 0.7213. AUC: 0.834. Nomogram model: C-index: 0.851.	CONUT score could increase the predictability of recurrence.
Suarez-Ibarrola et al. [106]	Predicting recurrence rates in 3 years from clinicopathological markers for patients undergoing TURBT—**547 patients.**	**Clinicopathological:** 3 significant markers including 2 from the surgical checklist—the number of tumors, the location of the tumors, and the intravesical therapy.	Statistical analysis only (Cox regression model).	(*p* < 0.05) considered to be significant.	The high quality of TURBT could drastically enhance the prediction of recurrence rates.
Li, S. et al. [107]	Predicting recurrence in 2 years from clinicopathological markers after different operative methods: (pin-ERBT ^6^, TURBT, and HoLRBT ^7^)—**115 patients**.	**Clinicopathological:** 4 markers including age, operative method, smoking, and tumor grade.	Statistical approach only.	(*p* < 0.05) considered to be significant. Recurrence rate for each operative method was: pin-ERBT: 0.10. TURBT: 0.385. HoLRBT: 0.40.	Pin-ERBT can decrease the risk of recurrence comparing to TURBT and HoLRBT.
Ajili, F. et al. [108]	Prediction of recurrence after BCG immunotherapy from clinicopathological and genomics markers. **308 patients.**	**Clinicopathological:** 8 markers including age, gender, tumor stage and grade, carcinoma in situ, size of tumor, multiplicity, and smoking. **Genomics:** A single marker: CD34 expression. **Combined:** 9 markers (genomics + clinicopathological).	MLP ^2^ based ANN	***Testing:*** **Clinicopathol-ogical:** - **Genomics:** - **Combined:** Acc: 0.975. Sen: 0.966. Spe: 1.00.	ANN model promises a good performance for predicting recurrence with combined genetic clinicopathological markers.
Zhan. Y. et al. [109]	Prediction of recurrence from urinary markers using three lncRNAs ^5^ panel: (MALAT1, PCAT-1, and SPRY4-IT1)—**368 patients**.	**Genomic:** 3 markers: MALAT1, PCAT-1, and SPRY4-IT1. **Combined:** 4 markers including previous 3 urinary markers and tumor stage.	**Genomic:** logistic regression. **Combined:** Statistical analysis only (Cox regression).	***Validation:*** **Genomic:** Sen: 0.625. Spe: 0.85. AUC: 0.813. **Combined:** *p* < 0.05 considered to be significant.	Urinary exosomal panels can effectively predict recurrence. Furthermore, PCAT-1 can independently predict recurrence.
Gogalic, Selma et al. [110]	Prediction of current recurrence using combined markers—**45 patients.**	**Genomics:** 5 markers including ECadh, IL8, MMP9, EN2, and VEGF with adjusting creatinine levels. **Clinicopathological:** 3 markers including tumor stage, No. of past recurrences and No. of BCG therapies). **Combined:** 8 markers including aforesaid markers.	LASSO logistic regression.	***Validating:*** **Genomics:** AUC: 0.75. **Clinicopathol-ogical:** AUC: 0.72. **Combined:** AUC: 0.84.	Combined markers model outperformed any individual markers models, especially after adjusting creatinine levels.
López de Maturana, E. et al. [111]	Prediction of recurrence in 4 years from clinicopathological and genomics markers—**995 patients**	**Clinicopathological:** 6 markers including area, gender, No. of tumors, tumor stage and grade, tumor size, and undergoing treatment. **Genomics:** 171,295 SNP markers. **Combined:** 171,301 markers (genomics + clinicopathological).	Statistical models only: **Clinicopathol-ogical:** Bayesian sequential threshold model. **Genomics:** markers selection: $R^{2}$ < 0.2. Prediction: Bayesian LASSO. **Combined:** Bayesian sequential threshold model coupled with LASSO.	***Testing:*** **Clinicopathol-ogical:** AUC: 0.62. $R^{2}$: 0.031. **Genomics:** AUC: 0.55. $R^{2}$: 0.010. **Combined:** AUC: 0.61. $R^{2}$: 0.041.	Genomics markers did not improve the model predictability for recurrence.

^1^ SVM-based recursive feature elimination; ^2^ multilayer perceptron; ^3^ this scoring system includes serum albumin, total lymphocyte count, and total cholesterol; ^4^ upper urinary tract urothelial carcinoma; ^5^ long non-coding RNAs; ^6^ transurethral en bloc resection of bladder tumor by pin-shaped electrode; ^7^ transurethral holmium laser resection of bladder tumor.
